# Characterization and Analytical Method Validation for Potential Impurities of a Merchantability Drug Substance Fluoxetine HCl

**DOI:** 10.1002/bmc.6069

**Published:** 2025-01-02

**Authors:** Rajani Reddy Janna Reddy, Sunder Kumar Kolli, Suresh Salakolusu, Sudha Divya Madhuri Kallam, Jayaprakash Kanijam Raghupathi, Naresh Kumar Katari

**Affiliations:** ^1^ Department of Chemistry BEST Innovation University Gorantla Andhra Pradesh India; ^2^ Department of Chemistry Annamacharya Institute of Technology & Sciences Hyderabad India; ^3^ Analytical Discovery Chemistry Aragen Life Sciences Pvt. Ltd. Hyderabad India; ^4^ Research and Development HIKMA Pharmaceuticals Columbus Ohio USA; ^5^ Department of Food Science Purdue University West Lafayette Indiana USA; ^6^ School of Chemistry & Physics College of Agriculture, Engineering & Science, Westville Campus, University of KwaZulu‐Natal Durban South Africa

**Keywords:** fluoxetine hydrochloride, method validation, nuclear magnetic resonance spectroscopy, reverse phase HPLC

## Abstract

A new selective and sensitive high‐performance liquid chromatography (HPLC) method was developed for the quantification of potential impurities in fluoxetine hydrochloride. Chromatographic separation was achieved on an end‐capped octadecylsilyl silica gel (Gemini‐C18 150 mm × 4.6 mm, 3.0 μm) using a gradient program with triethylamine, methanol, and water as the mobile phase at a flow rate of 1.0 mL/min and monitored at 215 nm. The run time was 60 min. The method was validated to fulfill International Conference on Harmonization (ICH Q2(R2)) requirements, and this validation included specificity, precision, linearity, limit of detection (LOD), limit of quantification (LOQ), and accuracy. The calibration curve was linear over the concentration range from LOQ to 120% with respect to sample concentration. The accuracy of the method is within the acceptable limit of 80%–120%. The results obtained for all parameters were within the acceptance criteria. So, this method can be employed for the regular analysis of potential impurities in the fluoxetine hydrochloride API.

**Abbreviations**
API: active pharmaceutical ingredient
ESI: electrospray ionization
FLX: fluoxetine hydrochloride
ICH: International Council for Harmonization
NMR: nuclear magnetic resonance spectroscopy
PDA: photodiode array detector
TTC: threshold of toxicological concern
SSRI: selective serotonin reuptake inhibitor

## 1 Introduction

The major regional pharmacopeias and worldwide accepted pharmacopeias like Europe and the United States provide common quality reference standards throughout the pharmaceutical industry to control the quality of medicines and the substances (API) used to manufacture them. These reference standards only give information about impurities that are reported in corresponding pharmacopeia, but not all the process‐related impurities. Not always process‐related impurities will be the same as reported in the pharmacopeia because synthetic routes are different from one another, and these impurities are considered unspecified impurities. For these process‐related impurities, it needs to develop the in‐house method and be reported as unspecified. As per EP and USP, generally specified impurities are with a limit of not more than 0.15% and unspecified impurities: not more than 0.10% [International Council for Harmonisation Guideline on Q2 (R1) Validation of Analytical Procedures 2005].

HPLC and gas chromatography (GC) are the most accurate analytical tools used for both quantitative and qualitative to identify related substances, process impurities, degradation impurities, residual solvents, inorganic contaminants, genotoxic, and nitrosamines up to PPB levels. Today's technology is developing very rapidly in finding the impurities in drug substances. Earlier, only potency was defined for a drug. Long‐lasting usage of a drug might lead to side effects if any impurities are detected in the drug. So, these impurities should be within the limit to release a drug substance. The development of specific LC (liquid chromatography) or GC methods for the determination of those impurities at the trace level is a complex process.

Detection, identification, and quantitation of impurities in drug substances are a significant part of drug development. Generally, most of the specified and unspecified impurity limits are defined based on ICH guidelines. In cases of genotoxic impurities and nitrosamines, limits are defined based on the maximum daily dose or sometimes on the worst case. Based on the threshold of toxicological concern (TTC) concept (1.5 μg/day for lifetime exposure) [International Council for Harmonisation Guideline on M7(R1) 2018], the development of such accurate methods is challenging to identify and quantify the impurities at trace level.

To identify and quantify the process‐related impurities, we have chosen fluoxetine hydrochloride as a drug substance. Fluoxetine hydrochloride is the most popular drug used as an antidepressant and is known as a selective serotonin reuptake inhibitor (SSRI) [American Society of Health‐System Pharmacists, Fluoxetine hydrochloride 2015]. It is often used to treat depression and sometimes obsessive–compulsive disorder and bulimia. The molecular formula and molecular weight of fluoxetine hydrochloride are C_17_H_18_F_3_NO.HCl and 345.8 g/mol [U.S. Pharmacopeial Convention. n.d.. *Fluoxetine hydrochloride*]. Many synthetic routes are available for the synthesis of fluoxetine hydrochloride [Matthias 2010; Tripathi 2012; Nageswara Rao and Nagaraju 2003; Martano et al. 2018]. According to Lukacs, one of the most widely used synthetic routes in these is listed below. In these one of the most popular synthetic route as given below which is reported by Lukacs [Mezei 2010].

**FIGURE 1:**
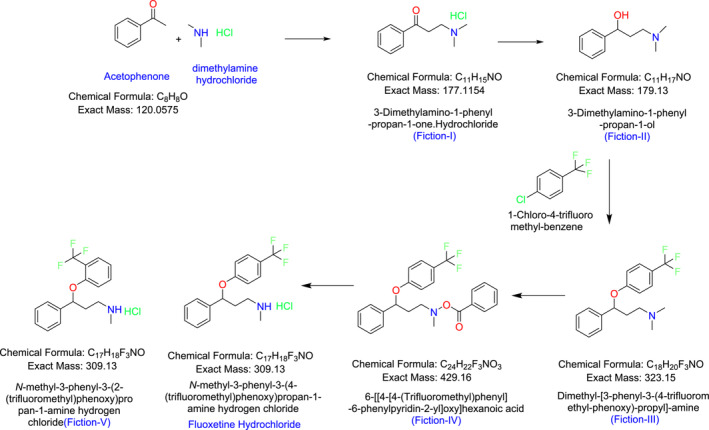
Synthesis of fluoxetine hydrochloride.

The fluoxetine hydrochloride ((3RS)‐N‐methyl‐3‐phenyl‐3‐[4‐tiflouromethyl) phenoxy]‐propan‐1‐amine hydrochloride) route of synthesis and its potential impurities (Figure 1).

Methods and specifications adopted for fluoxetine hydrochloride (API) have been revised several times for better testing quality in EP. Present revised specifications as per EP for specified impurities are Impurity‐A ((1RS)‐3‐(methylamino)‐1‐phenylpropan‐1‐ol) and Impurity‐B (N‐methyl‐3‐phenylpropan‐1‐amine) with a limit of NMT 0.15% and unspecified impurities: NMT 0.10% [European pharmacopeia 10.3 version. Monograph number: 01104. 2020; United States Pharmacopeial Convention. *Chromatography* 2023].

However, the abovementioned process‐related impurities are not stated in any pharmacopeia like EP and USP. Many analytical methods have been reported for the determination of fluoxetine hydrochloride pure drug [Clementina, Alexandra, and Valentina 2018; Zaheer et al. 2010; Naik, Prasada Rao, and Dhachinamoorthi 2018; Hire, Aher, and Bachhav 2023; Wassel and El‐agezy 2021]. Most of the analytical methods were carried out by HPLC or GC for impurities specified in EP and USP. Hence, we tried to develop a single method for the estimation of carryover impurities in fluoxetine hydrochloride by using proper system suitability methods [Indian Pharmacopoeia Commission. *Validation of analytical methods*. 2021; Wirth et al. 1997]. Developing each method on an individual basis would be incredibly time‐ and resource‐consuming for pharmaceutical companies. But, based on the route of synthesis, some impurities may not be detected in a single method. So, we developed a simple and single method by using universally available instruments (HPLC with UV detector), columns, and chemicals. Known compounds, which are process‐related and possible‐related impurities, toxicity is tested by using the Nexus2.5 tool and found no toxic alert [Ashby and Tennant 1988; Ashby and Paton 1995; Fetterman et al. 1997; Dobo et al. 2006; Snyder 2009], and the results are mentioned in Table 1.

**TABLE 1:** Toxicity prediction of fluoxetine impurities.

Structure	ICH M7 class	Cohort of concern	Derek prediction	Sarah prediction	Experimental data	Similarity to API	Overall in silico
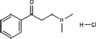 Fiction‐I	Inconclusive	No	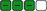	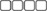	Carc: Unspecified Ames: Unspecified	No Derek alerts found	Inconclusive
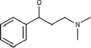 Fiction‐II	Class 5	No	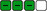	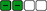	Carc: Unspecified Ames: Unspecified	No Derek alerts found	Negative
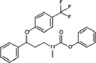 Fiction‐III	Class 5	No	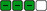	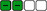	Carc: Unspecified Ames: Unspecified	No Derek alerts found	Negative
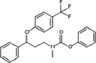 Fiction‐IV	Class 5	No	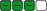	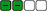	Carc: Unspecified Ames: Unspecified	No Derek alerts found	Negative
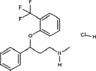 Fiction‐V	Class 5	No	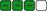	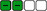	Carc: Unspecified Ames: Unspecified	No Derek alerts found	Negative

The optimized method is validated based on the ICH guidelines. So quantification was found with a specification limit of not more than 0.15% with respect to the test solution, and it covers parameters like specificity and system suitability, method precision, limit of detection (LOD) and limit of quantification (LOQ) establishment, LOQ precision, accuracy, and linearity. Quantified impurities are 3‐dimethylamino‐1‐phenyl‐propan‐1‐one hydrochloride (Fiction‐I), 3‐dimethylamino‐1‐phenyl‐propan‐1‐ol (Fiction‐II), dimethyl‐[3‐phenyl‐3‐(4‐trifluoromethyl‐phenoxy)‐propyl]‐amine (Fiction‐III), methyl‐[3‐phenyl‐3‐(4‐trifluoromethyl‐phenoxy)‐propyl] carbanic acid phenyl ester (Fiction‐IV), and 3‐(2‐(trifluoromethyl)phenoxy)‐N‐methyl‐3‐phenylpropan‐1‐amine hydrochloride (Fiction‐V).

## 2 Characterization by NMR

**FIGURE 2:**
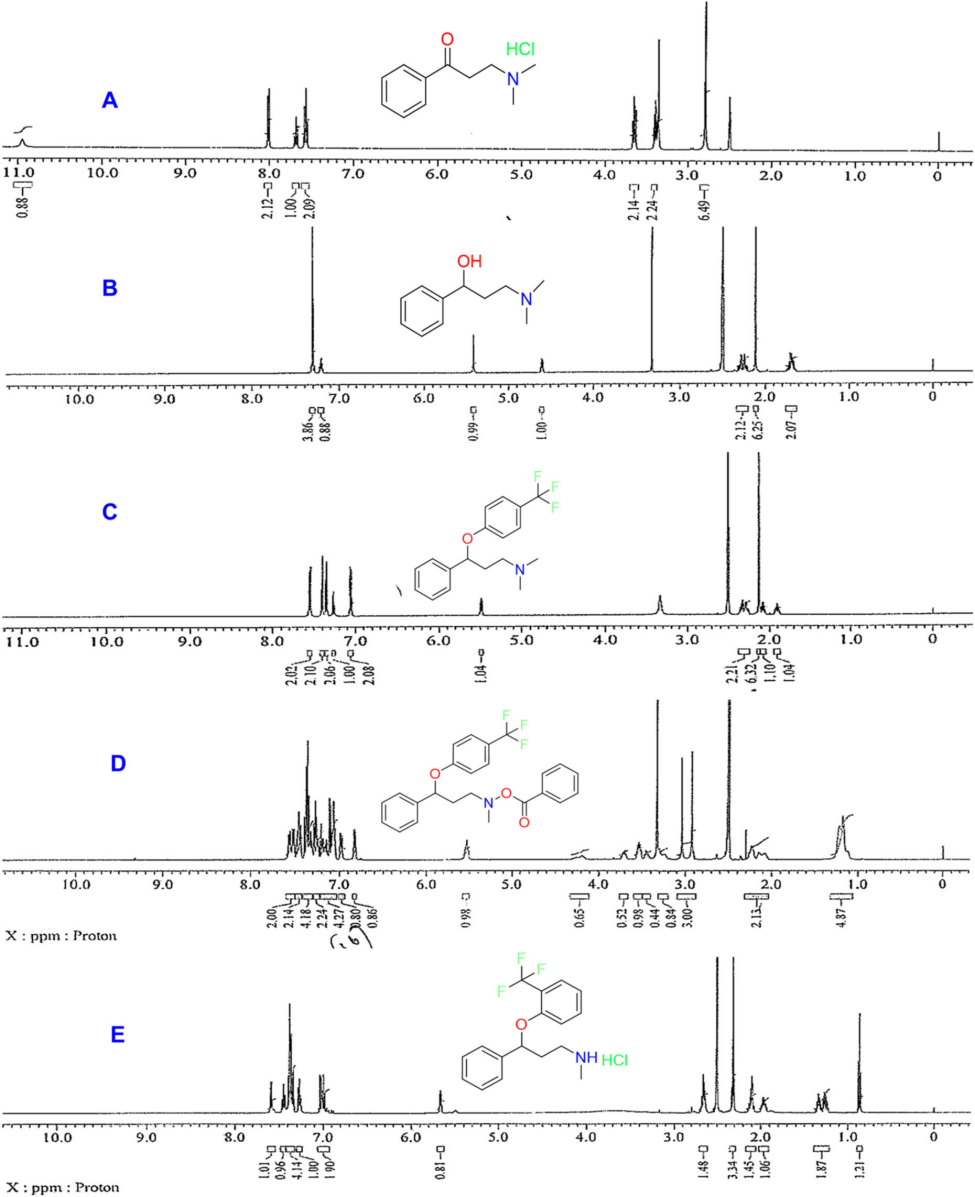
^1^H NMR spectrums of fluoxetine impurities: Fiction‐I (A), Fiction‐II (B), Fiction‐III (C), Fiction‐IV (D), and Fiction‐V (E).

Proton NMR has recorded all five impurities in DMSO as additional data. The results are mentioned below. In that, all the structures are confirmed as per proton count and proton environments. The peak‐splitting pattern also matches the respective neighboring protons (Figure 2).

## 3 Materials and Methods

### 3.1 Chemicals and Reagents

Fluoxetine hydrochloride and its impurities are procured from R&D, Nosch Labs, Veliminedu, Telangana. Triethylamine, orthophosphoric acid, and methanol are procured from Merck Chemicals (India).

### 3.2 Instruments

The proposed study was achieved by using a reverse‐phase HPLC system (Shimadzu LC‐2010C) that consisted of an LC furnished with a PDA detector. Chromatographic separations were done on an end‐capped octadecylsilyl silica gel (Gemini‐C18) (150 mm × 4.6 mm, 3.0 μm) column, an electronic balance (Shimadzu), pH meter (LAB India), Nexus, Derek Nexus, Meteor Nexus, Sarah Nexus, and Vitic Link software and NMR‐400MHz (JEOL).

### 3.3 Chromatographic Method Conditions

The analysis of fluoxetine hydrochloride employed the Gemini C18 (150 mm × 4.6 mm, 3.0 μm). A gradient elution technique was utilized with a mobile phase solution consisting of a Mobile Phase‐A with methanol and buffer solution in the ratio 20:80 v/v. The buffer solution was prepared by mixing 12.5 mL of triethylamine and about 900 mL of water. Adjust the pH to 6.0 with phosphoric acid and dilute to 1000 mL with water. Mobile Phase‐B as 100% methanol. Gradient time (min)/B conc (%) is as follows: 0/25, 2/25, 2.1/44, 20/44, 30/80, 45/80, 50/44, 55/44, 55.1/25, and 60/25. The column temperature was set to 30°C, and the flow rate of the mobile phase was 1.0 mL min^−1^. The injection volume was 10 μL, and the detection wavelength was optimized at 215 nm. The diluent was a 40:60 v/v ratio of methanol and buffer solution. The sample concentration is 20 mg/mL.

### 3.4 Preparation of Impurity Stock Solution

#### 3.4.1 0.25 mg/mL of Each Impurity With Solvent Mixture

Weigh about 5 mg of Fiction‐I, 5 mg of Fiction‐II, 5 mg of Fiction‐III, 5 mg of Fiction‐IV, and 5 mg of Fiction‐V into a 20 mL volumetric flask, dissolve, and dilute to volume with the solvent mixture.

#### 3.4.2 Test Sample Preparation

Weigh and dispense approximately 500 mg of the substance to be examined into a 25‐mL volumetric flask. Then, introduce 10 mL of diluent, subject it to sonication to dissolve, and make up to volume with diluent and mix well.

### 3.5 Development and Validation of HPLC Method

**FIGURE 3:**
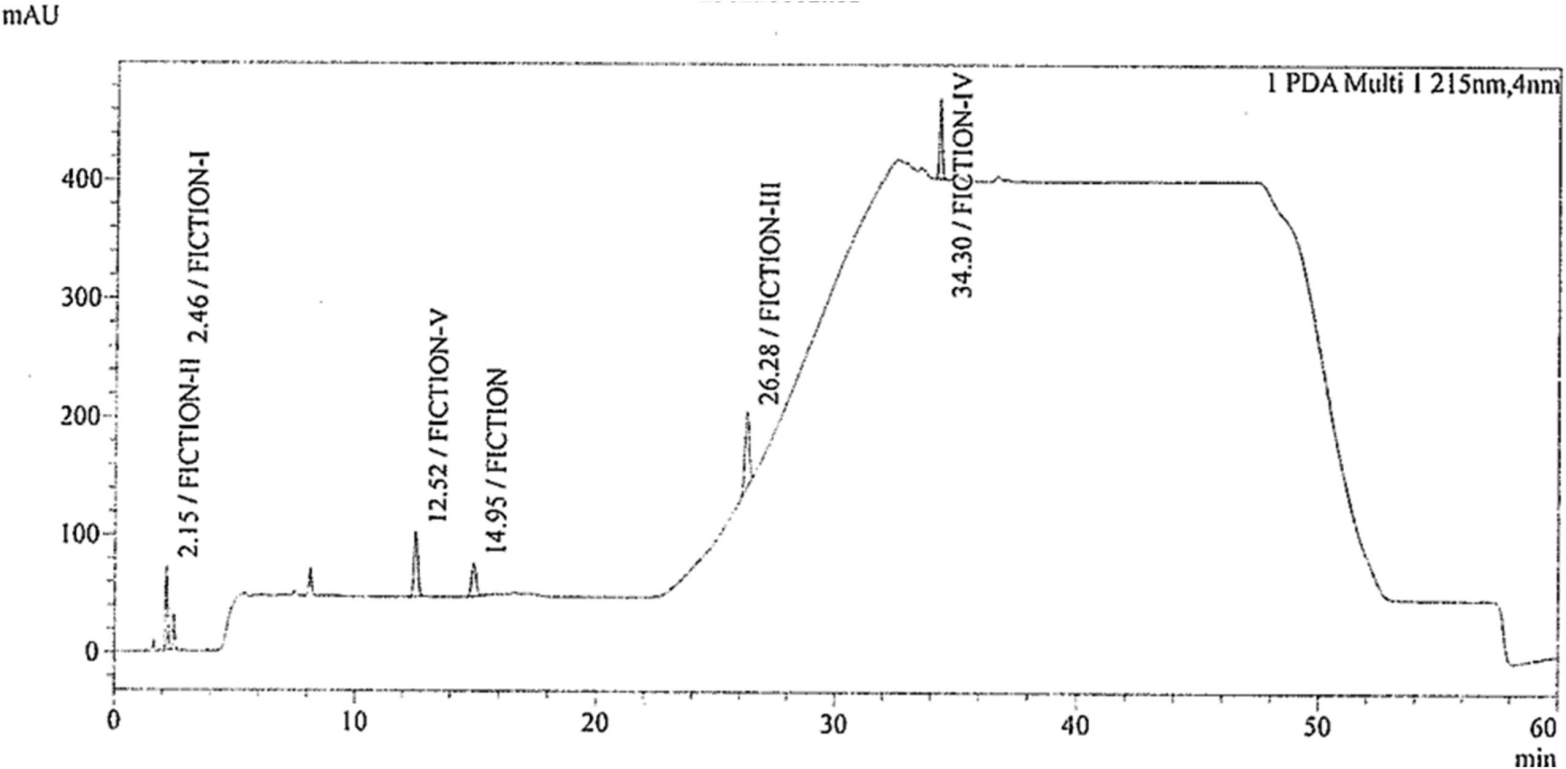
HPLC chromatogram of fluoxetine and its related impurities.

As the selected impurities are mixtures of polar and nonpolar, the method trial was conducted on reverse‐phase HPLC due to high resolution, sensitivity, and good repeatability. The study was performed to develop an HPLC quantitative method to quantify the potential impurities. Initially, the selection of columns, mobile phase composition, and adjustment of gradient program time were done by screening. At this time observed that the Fiction‐I solution should be prepared freshly before injection. After getting the preliminary data, the experimental work was carried out by a gradient program at a flow rate of 1.0 mL/min, which is mentioned in Table 2, and the HPLC trace is shown in Figure 3.

**TABLE 2:** Optimized HPLC method conditions.

Column	Gemini C18 (150 mm × 4.6 mm, 3.0 μm)
Buffer	Mix 12.5 mL of triethylamine and about 900 mL of water Adjust the pH to 6.0 with phosphoric acid and dilute to 1000 mL with water
Mobile Phase‐A	Methanol and buffer solution in the ratio 20:80 v/v
Mobile Phase‐B	100% methanol
Flow	1.0 mL/min
Injection volume	10 μL
Detection	UV at 215 nm
Column temperature	30°C
Elution	Gradient time (min)/B con (%): 0/25, 2/25, 2.1/44, 20/44, 30/80, 45/80, 50/44, 55/44, 55.1/25, and 60/25
Run time	60 min
Diluent	Methanol:buffer (40:60% v/v)
Test concentration	20 mg/mL

The run time obtained for Fiction‐I is 2.4, Fiction‐II is 2.1, Fiction‐III is 26.2, Fiction‐IV is 34.2, and Fiction‐V is 12.5 after optimizing the method. Then, the method was validated as per the International Conference on Harmonization guidelines. The suitability of the method was verified by estimating different parameters, such as system suitability, specificity, precision, LOD, LOQ, accuracy, and linearity.

## 4 Results and Discussion

The present study was to validate a method for quantification of potential impurities in fluoxetine hydrochloride. The validation was carried out by making a standard solution of 20 mg/mL of fluoxetine hydrochloride. The impurities were quantified with a limit of 0.15% with respect to the sample solution (fluoxetine hydrochloride).

### 4.1 System Suitability

The method suitability was checked by a resolution of not less than 1.5 between Fiction‐I and Fiction‐II peaks from the standard solution, which contains all impurity standards. And peaks are separated with a result of 1.8 resolution, which are mentioned in Table 3 and Table S1.

**TABLE 3:** Method validation summary for fluoxetine hydrochloride and its related impurities.

Impurity name	Fiction‐I	Fiction‐II	Fiction‐III	Fiction‐IV	Fiction‐V
Retention time (minutes)	2.46	2.15	26.27	34.26	12.57
System suitability	1.8	—	32.7	30.6	42.6
Linearity correlation coefficient (*R*)	0.994	0.998	0.995	0.996	0.996
Intercept	4164.96	1078.78	82569.15	5302.10	7699.61
Slope	4722.95	13530.25	21154.67	21165.77	17502.75
Detection limit, LOD (%)	0.0252	0.0023	0.0007	0.0006	0.0051
Quantitation limit, LOQ (%)	0.076	0.007	0.002	0.002	0.015
LOQ precision, %RSD (*n* = 6)	5.75	0.40	0.22	1.32	0.50
Accuracy at LOQ level (*n* = 3), % average recovery	1.9	1.0	6.2	0.5	0.6
Accuracy at 80% level (*n* = 3), % average recovery	1.9	0.3	3.0	2.9	0.5
Accuracy at 100% level (*n* = 3), % average recovery	1.8	0.2	1.1	0.6	3.7
Accuracy at 120% level (*n* = 3), % average recovery	2.1	0.6	3.6	2.9	0.6
System precession(*n* = 6), %RSD	3.56	0.72	0.85	1.9	1.05
Method precession(*n* = 6), %RSD	4.42	0.48	0.5	1.65	2.09

### 4.2 Specificity

Specificity is the ability to assess unequivocally the analyte in the presence of components that may be expected to be present. Typically, these might include impurities, degradants, and matrix.

Retention times of impurities in spiked solution match with equal retention times in individual solutions. In blank no interference with retention times of known peaks, and the results are in Table 3.

### 4.3 Method Precision (Repeatability)

Repeatability should be assessed using a minimum of six determinations at 100% of the test concentration as per ICH guideline Q2(R2). Thus, the test of repeatability was verified on six different spiked samples with a target concentration of 100% impurity standard solution. The percentage of impurities from the spiked sample was calculated from six different spiked preparations that were used for precision evaluation, and the results are mentioned in Table 3 and Table S2.

The relative standard deviation determined from spiked solution in six individual injections should not be more than 5.0%, and the results met the specification.

### 4.4 LOD and LOQ Establishment

Three different concentrations of 0.05%, 0.10%, and 0.15% were prepared with respect to sample concentration and injected each concentration as a triplicate into the chromatograph. Established the limits based on the standard deviation of the response and the slope. The results are mentioned in Table 3.

### 4.5 Precision at LOQ Level Solution

The limit should be subsequently validated by the analysis of a suitable number of samples known to be near or prepared at the quantitation limit. A LOQ‐level solution has been prepared for Fiction‐I (0.07%), Fiction‐II (0.01%), Fiction‐III (0.05%), Fiction‐IV (0.01%), Fiction‐V (0.015%), and Fiction (0.02%) with respective sample concentrations, and six replicate injections were injected into the chromatograph and calculated the %RSD for the area counts. The results are mentioned in Table 3 and Table S3. The %RSD from the LOQ level solution for area response should be not more than 10.0, and the results met the specification limit.

### 4.6 Linearity

A linear relationship should be evaluated across the range of the analytical procedure. As per guidelines for the establishment of linearity, a minimum of five concentrations is recommended.

**FIGURE 4:**
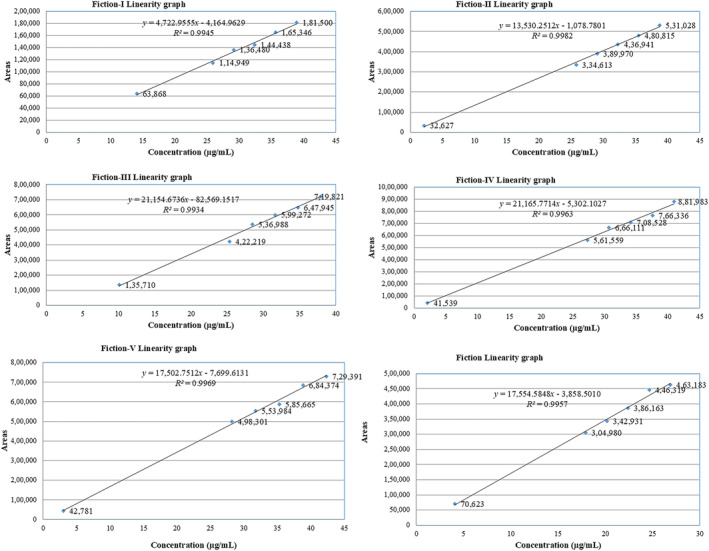
Linearity graph for all impurities.

A series of linearity solutions for the fiction and impurities from LOQ, 80%, 90%, 100%, 110%, and 120% of the target concentration have been prepared and injected into the chromatograph. The correlation coefficient results are mentioned in Figure 4 and Table 3. The correlation coefficient (*R* ^2^) (average area response vs. concentration) is not less than 0.990. And the results met the acceptance criteria. The results obtained in linearity are mentioned in Table 3.

### 4.7 Accuracy

The accuracy or trueness of an analytical procedure expresses the closeness of agreement between the value that is accepted either as a conventional true value or an accepted reference value and the value found.

The accuracy has been verified by spiking the sample at four different concentrations, namely, LOQ, 80%, 100%, and 120%. At each level, triplicate individual preparations were injected into the chromatograph. These samples were analyzed along with unspiked samples. The percentage recoveries were calculated, and the results are mentioned in Table 3 and Table S4.

In the specificity parameter, spiked sample solution along with its related impurities was injected into the chromatograph derived the retention times, and no variation has been observed between spiked sample RT and individual impurities RT. Impurities were well separated from the fiction in the spiked sample, and peak purity results were within the acceptance criteria.

The method precision was carried out by spiking impurities into six different samples at the specification level. The precision evaluation data met the acceptance criteria. In the LOD and LOQ establishment parameter, three different concentrations, namely, 0.05%, 0.10%, and 0.15% were prepared with respect to the 100% sample concentration and injected each concentration as a triplicate into the chromatograph and established the LOD and LOQ levels. The LOQ‐level solution has been prepared for Fiction‐I (0.076%), Fiction‐II (0.007%), Fiction‐III (0.002%), Fiction‐IV (0.002%), and Fiction‐V (0.015%) with respective sample concentrations and obtained %RSD met acceptance criteria. A linearity study has been verified ranging from LOQ to 120% of the target concentration derived, and the correlation coefficient is 0.99. The accuracy study has been verified by injecting LOQ spiked, 80% spiked, 100% spiked, and 120% spiked solutions and calculating the percentage recovery is within 80%–120%. These results indicate that the proposed method is accurate.

## 5 Conclusion

The present study was conducted to establish a new HPLC analytical method for the quantification of potential impurities in fluoxetine hydrochloride. The method is simple, precise, and accurate. The developed method was validated as per ICH guidelines as cost‐effective, less time‐consuming, and a single method to quantify all potential impurities. All the validation parameters were found within the acceptance limit as per ICH guidelines. So that this method can be used for testing potential impurities in fluoxetine hydrochloride for routine analysis. In this method, we used methanol for the analysis, which is a cost‐effective and ecofriendly than most regular HPLC solvents.

## Conflicts of Interest

The authors declare no conflicts of interest.

## Supporting information


**Table S1.** Retention times of fiction and its impurities in individual and spiked solution.
**Table S2.** Results of impurities from spiked solution in six individual preparations.
**Table S3.** The %RSD area counts from six replicate injections of LOQ‐level solution.
**Table S4.** The percentage recovery result.
