# Supplementary material for: Characterization and Analytical Method Validation for Potential Impurities of a Merchantability Drug Substance Fluoxetine HCl
Source: Biomed Chromatogr. 2025 Jan 2;39(2):e6069. doi: 10.1002/bmc.6069 (PMC11695798; doi:10.1002/bmc.6069)

**Characterisation and Analytical method validation for potential impurities of a merchantability drug substance Fluoxetine HCl**

Rajani Reddy Janna Reddy^a^, Sunder Kumar Kolli^a,b*^, Suresh Salakolusu^c^, Sudha Divya Madhuri Kallam^d^, Jayaprakash Kanijam Raghupathi^e^, Naresh Kumar Katari^f*^

*^a^ Department of Chemistry, BEST Innovation University, Gownivaripalli, Gorantla, Andhra Pradesh-515231, INDIA.*

*^b^ Department of Chemistry, Annamacharya Institute of Technology & Sciences, Hyderabad 501512, INDIA.*

*^c^Analytical Discovery Chemistry, Aragen Life Sciences Pvt. Ltd., IDA Nacharam, Hyderabad, 500076, India.*

*^d^ Research and Development, HIKMA Pharmaceuticals, 1809 N Wilson Rd, Columbus, OH 43228, USA.*

*^e^Department of Food Science, Purdue University, West Lafayette, Indiana, USA- 47906.*

^f^ School of Chemistry & Physics, College of Agriculture, Engineering & Science, Westville Campus, University of KwaZulu-Natal, P Bag X 54001, Durban-4000, South Africa

*Corresponding Author: E-mail: [sunderkolli@gmail.com](mailto:sunderkolli@gmail.com); [dr.n.k.katari@gmail.com](mailto:dr.n.k.katari@gmail.com); KatariN@ukzn.ac.za

**Fiction-I**

**
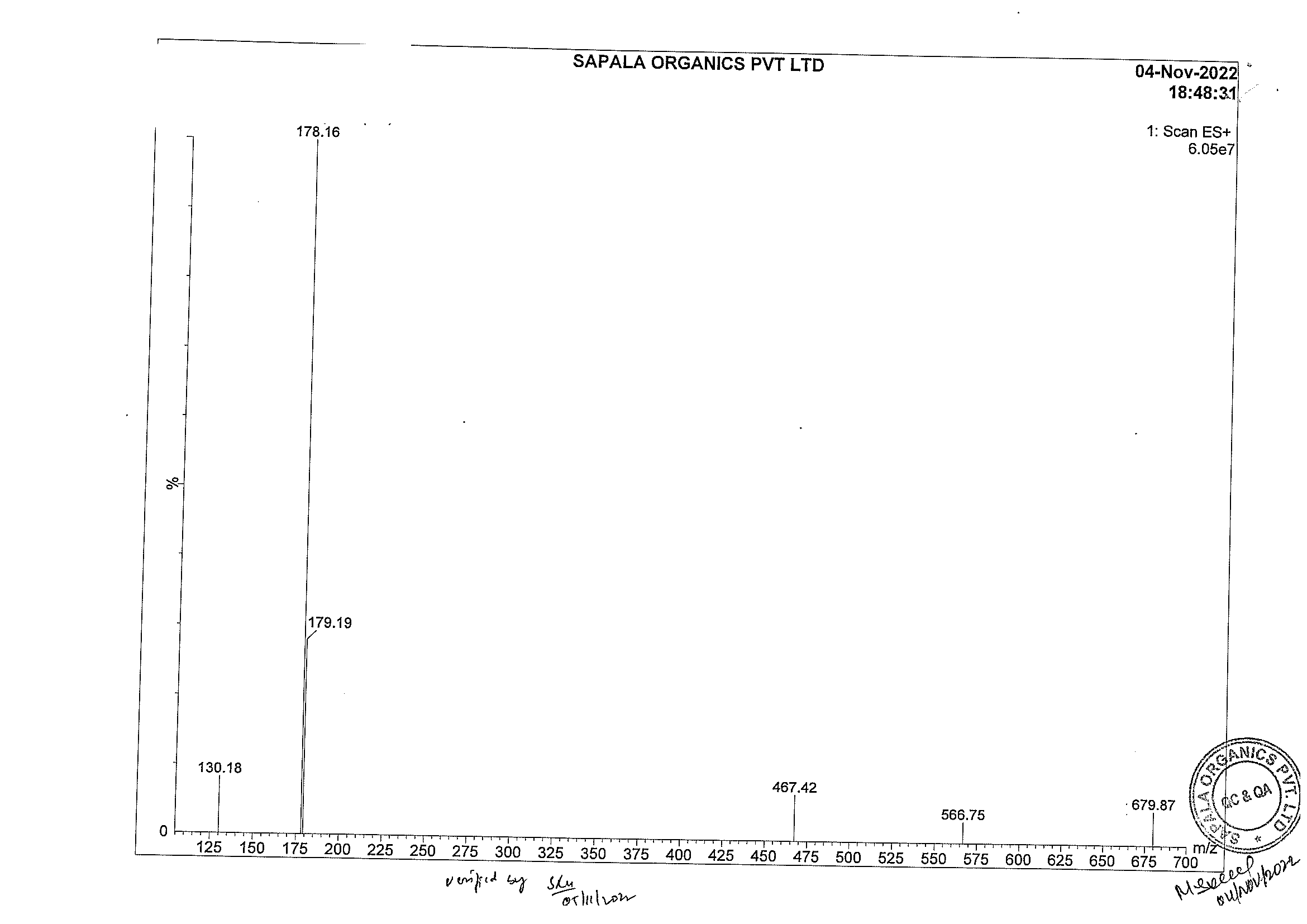
**

**
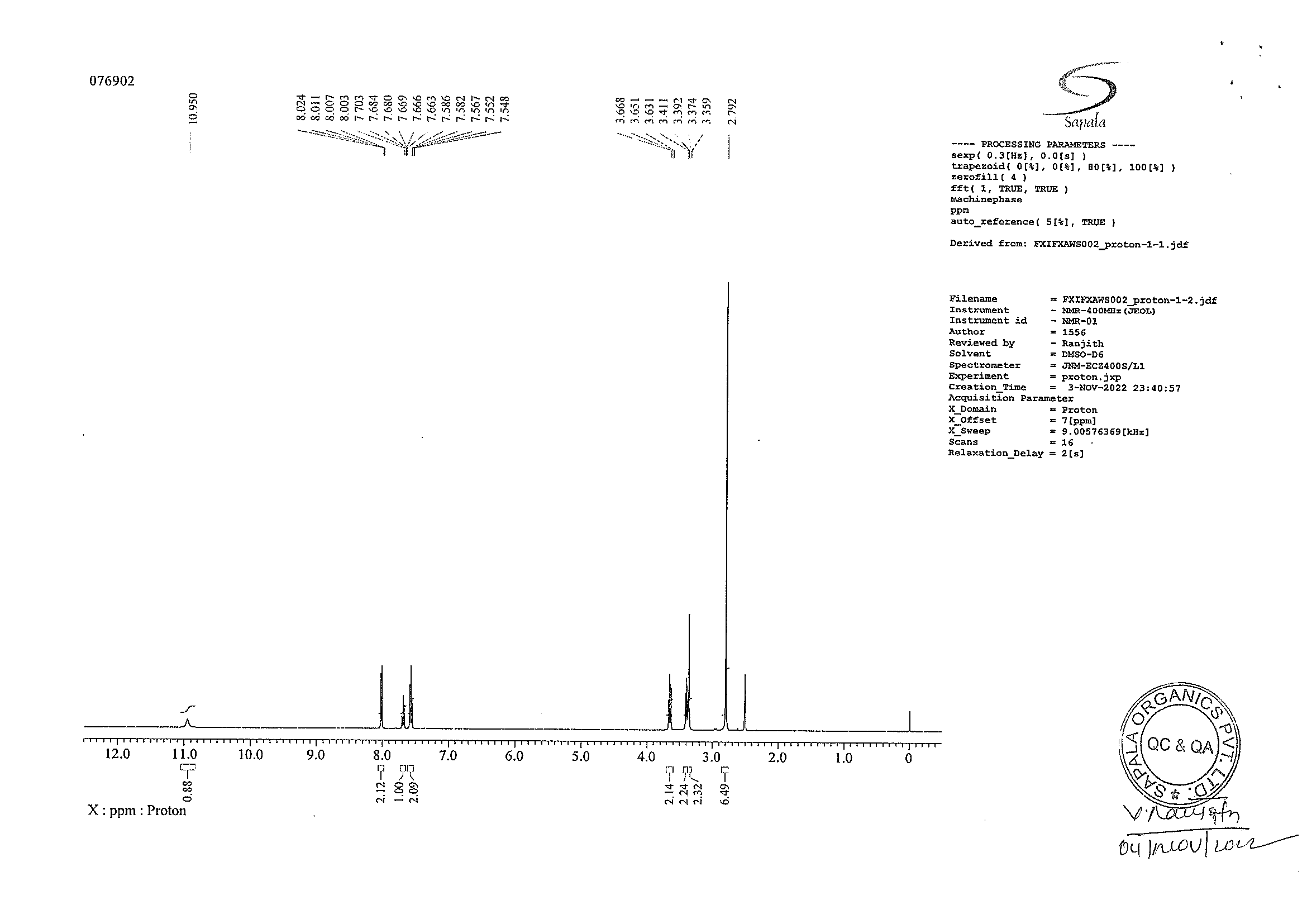
**

**Fiction-II**

**
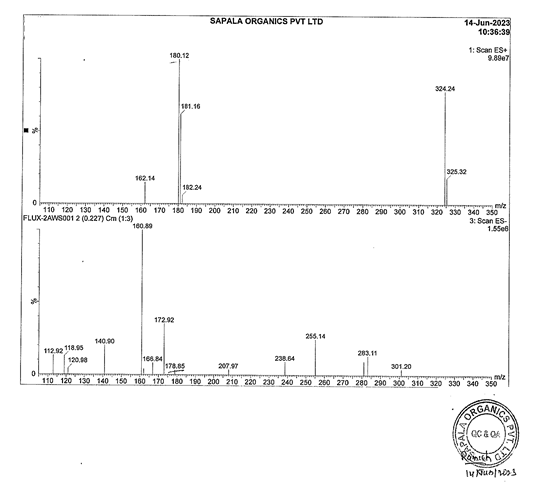
**

**
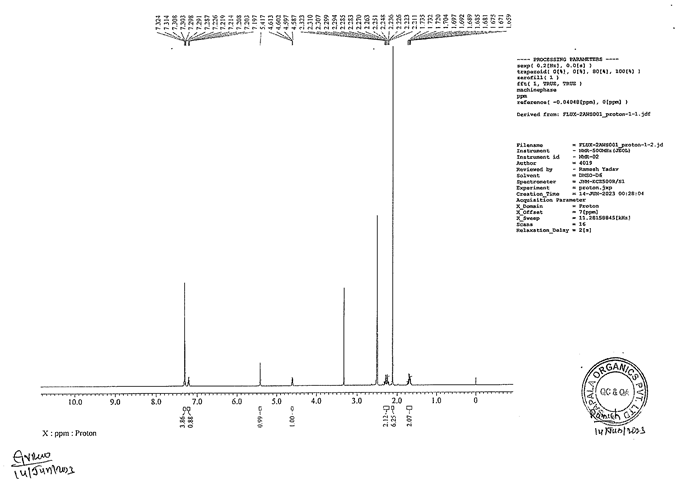
**

**Fiction-III**

**
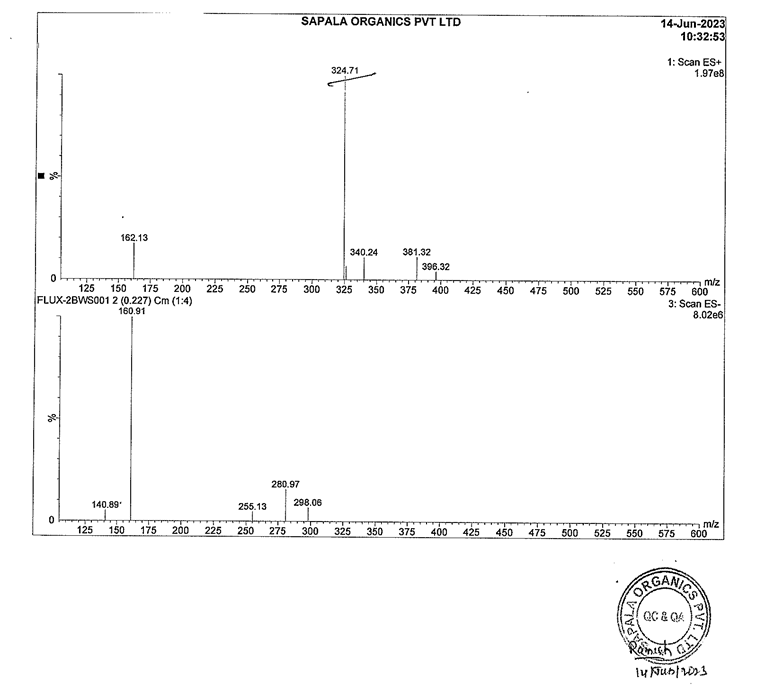
**

**
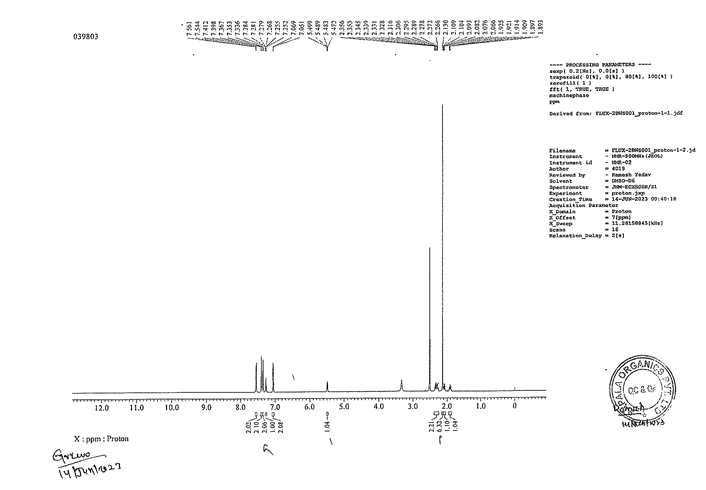
**

**Fiction-IV**

**
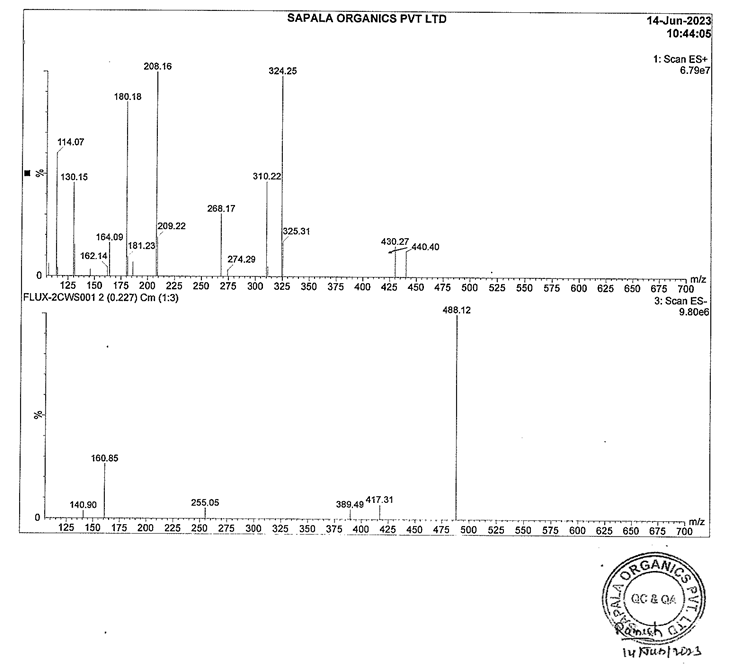
**

**
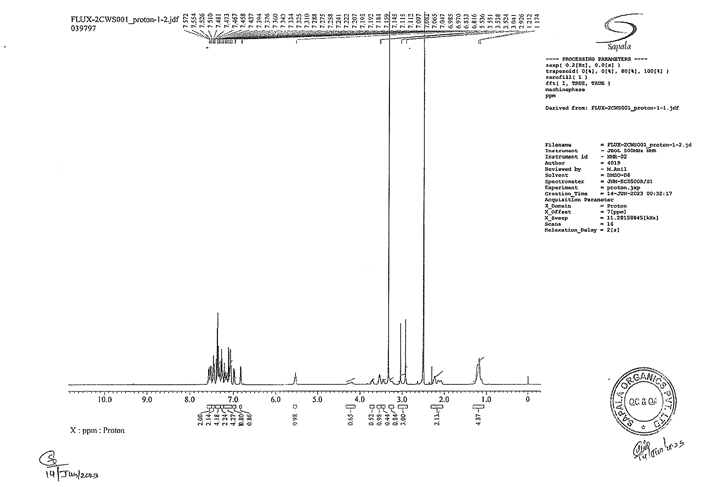
**

**Fiction-V**

**
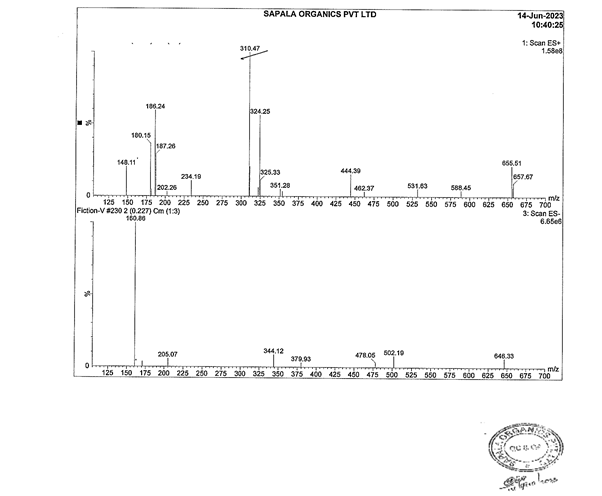
**

**
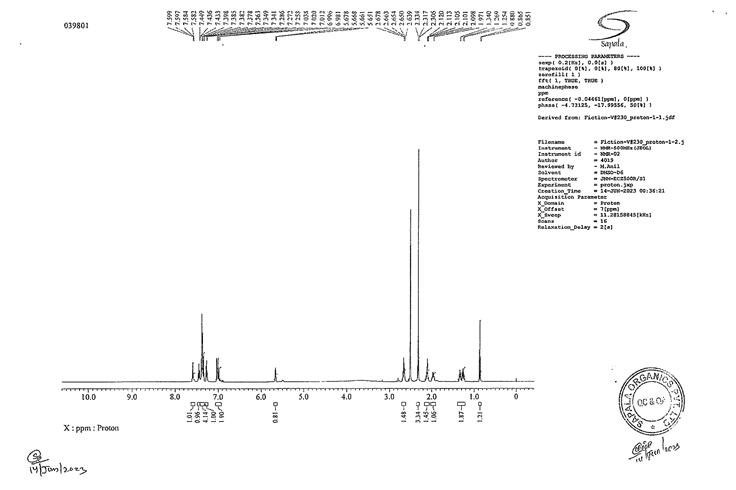
**

**Validation results:**

| **Name of the Impurity** | **Retention times** | |
| --- | --- | --- |
|  | **Individual solution** | **Spiked solution** |
| Fiction-I | 2.47 | 2.46 |
| Fiction-II | 2.15 | 2.15 |
| Fiction-III | 26.25 | 26.27 |
| Fiction-IV | 34.24 | 34.26 |
| Fiction-V | 12.56 | 12.57 |

*Table S 1 Retention times of Fiction and it’s impurities in individual and spiked solution.*

| **No. of Prep.** | **Fiction -I** | **Fiction -II** | **Fiction –III** | **Fiction -IV** | **Fiction –V** |
| --- | --- | --- | --- | --- | --- |
| 1 | 0.14 | 0.15 | 0.20 | 0.14 | 0.14 |
| 2 | 0.13 | 0.15 | 0.20 | 0.13 | 0.15 |
| 3 | 0.13 | 0.15 | 0.20 | 0.13 | 0.15 |
| 4 | 0.13 | 0.15 | 0.20 | 0.13 | 0.15 |
| 5 | 0.13 | 0.15 | 0.20 | 0.14 | 0.15 |
| 6 | 0.12 | 0.15 | 0.20 | 0.14 | 0.15 |
| **Average** | 0.13 | 0.15 | 0.20 | 0.14 | 0.15 |
| **Std. Dev** | 0.0057 | 0.0007 | 0.0010 | 0.0022 | 0.0032 |
| **%RSD** | 4.42 | 0.48 | 0.50 | 1.65 | 2.09 |

*Table S 2 Results of Impurities from spiked solution in six individual preparations.*

| **Area counts of** | | | | | | |
| --- | --- | --- | --- | --- | --- | --- |
| **SI. No** | **Fiction-I** | **Fiction-II** | **Fiction-III** | **Fiction-IV** | **Fiction-V** | **Fiction** |
| 1 | 108280 | 30469 | 186420 | 61829 | 61349 | 84742 |
| 2 | 104800 | 30141 | 185900 | 61047 | 61108 | 84763 |
| 3 | 101080 | 30477 | 185255 | 61696 | 61941 | 84641 |
| 4 | 99289 | 30357 | 185452 | 62774 | 61107 | 85082 |
| 5 | 95686 | 30403 | 185544 | 61598 | 61307 | 85423 |
| 6 | 92592 | 30397 | 185815 | 60346 | 61480 | 85294 |
| **Average** | 100288 | 30374 | 185731 | 61548 | 61382 | 84991 |
| **Std Dev** | 5766 | 123 | 412 | 813 | 310 | 323 |
| **%RSD** | 5.75 | 0.40 | 0.22 | 1.32 | 0.50 | 0.38 |

*Table S 3 The % RSD area counts from six replicate injections of LOQ level solution.*

| **% Recovery** | | | | | |
| --- | --- | --- | --- | --- | --- |
| **Name of the Conc.** | **Fiction-I** | **Fiction-II** | **Fiction-III** | **Fiction-IV** | **Fiction-V** |
| LOQ Pre-1 | 111.33 | 104.87 | 106.54 | 114.32 | 118.24 |
| LOQ Pre-2 | 109.13 | 102.84 | 99.02 | 113.34 | 116.87 |
| LOQ Pre-3 | 107.29 | 104.36 | 112.04 | 113.52 | 117.18 |
| **Average** | **109.3** | **104.0** | **105.9** | **113.7** | **117.4** |
| **Std.dev** | **2.0** | **1.1** | **6.5** | **0.5** | **0.7** |
| **%RSD** | **1.9** | **1.0** | **6.2** | **0.5** | **0.6** |
| 80% Pre-1 | 91.31 | 104.95 | 98.12 | 83.53 | 104.00 |
| 80% Pre-2 | 90.09 | 105.49 | 96.54 | 83.67 | 103.27 |
| 80% Pre-3 | 87.88 | 105.54 | 102.30 | 87.85 | 104.20 |
| **Average** | **89.8** | **105.3** | **99.0** | **85.0** | **103.8** |
| **Std.dev** | **1.7** | **0.3** | **3.0** | **2.5** | **0.5** |
| **%RSD** | **1.9** | **0.3** | **3.0** | **2.9** | **0.5** |
| 100% Pre-1 | 106.16 | 95.30 | 100.29 | 98.44 | 101.40 |
| 100% Pre-2 | 107.51 | 95.30 | 99.64 | 98.24 | 106.33 |
| 100% Pre-3 | 103.80 | 95.03 | 101.74 | 97.30 | 109.06 |
| **Average** | **105.8** | **95.2** | **100.6** | **98.0** | **105.6** |
| **Std.dev** | **1.9** | **0.2** | **1.1** | **0.6** | **3.9** |
| **%RSD** | **1.8** | **0.2** | **1.1** | **0.6** | **3.7** |
| 120% Pre-1 | 114.27 | 103.63 | 85.68 | 101.88 | 103.75 |
| 120% Pre-2 | 112.80 | 104.61 | 84.44 | 97.90 | 102.78 |
| 120% Pre-3 | 109.65 | 104.73 | 90.43 | 96.39 | 102.60 |
| **Average** | **112.2** | **104.3** | **86.9** | **98.7** | **103.0** |
| **Std.dev** | **2.4** | **0.6** | **3.2** | **2.8** | **0.6** |
| **%RSD** | **2.1** | **0.6** | **3.6** | **2.9** | **0.6** |

*Table S 4 The % Recoveries Result*


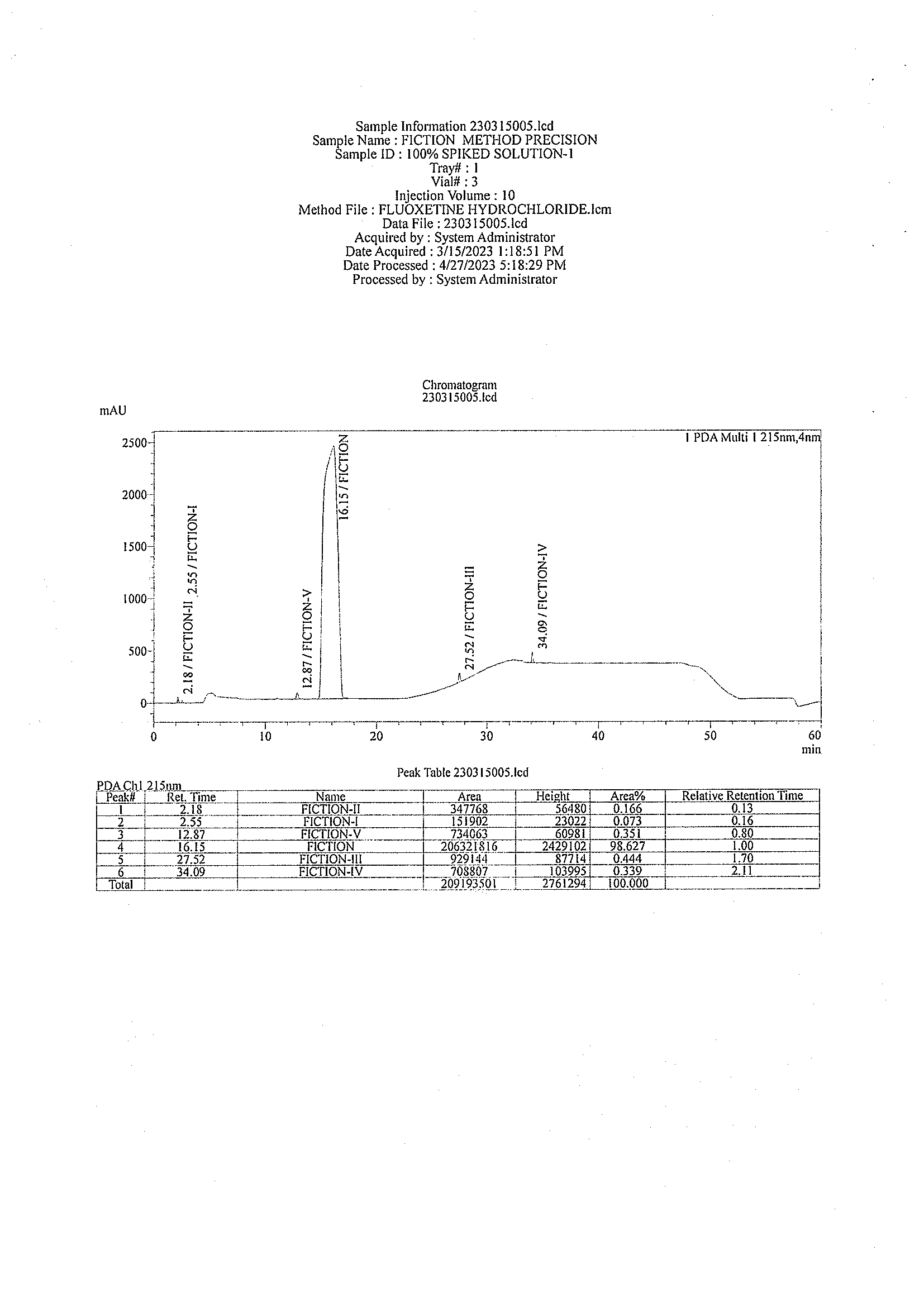

Supplement: Supplementary file 1 — Table S1. Retention times of fiction and its impurities in individual and spiked solution. Table S2. Results of impurities from spiked solution in six individual preparations. Table S3. The %RSD area counts from six replicate injections of LOQ‐level solution. Table S4. The percentage recovery result. [file BMC-39-e6069-s001.doc]
